# The Role of Metallodrugs in Enhancing Neuroendocrine Neoplasm Therapies: The Promising Anticancer Potential of Ruthenium-Based Complexes

**DOI:** 10.3390/molecules30183828

**Published:** 2025-09-21

**Authors:** Erika Stefàno, Federica De Castro, Asjad Ali, Michele Benedetti, Francesco Paolo Fanizzi

**Affiliations:** Department of Biological and Environmental Sciences and Technologies (DiSTeBA), University of Salento, Via Monteroni, I-73100 Lecce, Italy; erika.stefano@unisalento.it (E.S.); federica.decastro@unisalento.it (F.D.C.); asjad.ali@unisalento.it (A.A.); fp.fanizzi@unisalento.it (F.P.F.)

**Keywords:** metallodrugs, neuroendocrine neoplasms, ruthenium-based complexes, platinum-based chemotherapy, medicinal inorganic chemistry, IT-139 (KP-1339 or NKP1339)

## Abstract

Neuroendocrine neoplasms (NENs) represent a small and heterogeneous group of tumors that share a common phenotype, originating from cells within the endocrine and nervous systems. Metallodrugs have had a significant impact on the treatment of NENs, as platinum-based chemotherapy is the first-line therapy approved for managing these types of tumors. Currently, medicinal inorganic chemistry is investigating new metal-based drugs to mitigate the side effects of existing agents, including cisplatin and its derivative compounds. Among the emerging alternatives to platinum-based drugs, ruthenium-based complexes garnered attention as potential chemotherapeutics due to their notable antineoplastic and antimetastatic activity. This review focuses on the promising antitumor effects of certain Ru compounds in NEN therapy, emphasizing their potential in NEN treatment through interaction with new potential targets. Among these, IT-139 (also known as KP-1339 or NKP-1339), which has already entered clinical trials, and other new Ru compounds are highlighted.

## 1. Introduction

Metal-based drugs and imaging agents are widely used in clinical settings for the treatment and diagnosis of cancers and a variety of other diseases [1,2]. Compared to organic molecules, metal compounds possess unique structural features, varying charges, and additional electromagnetic properties. Their geometry (planar, octahedral, tetragonal pyramidal, etc.) depends on the type of metal center, oxidation state, and coordinated ligands, enabling diverse mechanisms of action, including (i) π-stacking (aromatic stacking) with base pairs and G-quadruplexes, incorporating end-stacking and intercalation (e.g., planar molecules), (ii) groove/loop binding, (iii) electrostatic interactions, (iv) direct coordination to nucleobases, and (v) phosphate backbone [3,4,5]. The central metal ions and appropriate ligand substituents confer cationic properties to the metal complex, enhancing electrostatic interactions with electronegative nucleic acids and cell permeability [6]. Furthermore, certain metal molecules possess optical, magnetic, or catalytic properties, enabling additional modes of action [4]. Cisplatin, the first and most used metal-based chemotherapy drug, was first synthesized in 1845 as Peyrone’s salt, but its anticancer activity was not discovered until 1965, when Barnett Rosenberg observed its ability to inhibit cell division. It is estimated that about 50% of all patients are treated with cisplatin for a broad range of solid tumors, including testicular, ovarian, bladder, lung, cervical, head and neck, gastric, and other types of cancer [7,8]. However, severe side effects and resistance phenomena are often observed following treatment with platinum drugs, prompting further research into new anticancer metallodrugs [9,10,11]. Among these, carboplatin and oxaliplatin, which represent the second- and third-generation platinum chemotherapy drugs, exhibit lower toxicity and reduced resistance to chemotherapy [12]. Other platinum agents have been approved in certain countries, such as nedaplatin (Japan), lobaplatin (China), heptaplatin (South Korea), and miriplatin (Japan) [13].

In addition to platinum-containing complexes, researchers have also explored other metals in the transition series (e.g., Ru(III), Ir(III), Pd(II), Au(I), Ti(IV), Cu(II), and Fe(II)), resulting in different mechanisms of action and antiproliferative properties [14,15,16]. Metal-based compounds have also revolutionized theranostics, an emerging field that combines diagnostics and therapeutics into single multifunctional formulations [17]. An example of this is the approval of new theranostic compounds, such as lutetium 177-based drugs [18], for use in gastroenteropancreatic neuroendocrine tumors. For high-grade NENs, the use of traditional agents, such as cisplatin, is still preferred [19,20].

This review aims to discuss the role of metallodrugs in NENs therapy, providing an update of currently approved metal-based drugs and promising drug candidates in pre-clinical and clinical studies. Specifically, we focus on Ru(II)- and Ru(III)-based complexes, which were intensively studied in recent decades and represent a viable alternative to cisplatin for the treatment of these types of tumors due to their therapeutic potential [21,22]. Among the ruthenium drugs currently in clinical trials, IT-139 (also known as KP-1339 or NKP-1339) has demonstrated effectiveness, particularly against carcinoid neuroendocrine tumors, including those resistant to different classes of anticancer agents [23]. To highlight both the challenges and opportunities offered by Ru anticancer drugs in neuroendocrine neoplasms, we also consider new Ru-based metallodrugs that have demonstrated promising anticancer properties in vitro.

## 2. Neuroendocrine Neoplasms: Classification and Treatment Options

Neuroendocrine neoplasms (NENs) are a heterogeneous group of malignancies that are considered rare due to their low incidence, constituting approximately ~2% of all malignancies [20]. However, registry studies have highlighted a global increase in the incidence of NEN [24]. The largest of these registries, the National Cancer Institute’s Surveillance, Epidemiology, and End Results (SEER) Program, has shown a rise in the incidence of gastroenteropancreatic neuroendocrine neoplasms (GEP-NEN)—specifically in the rectum, pancreas, and small intestine—from 1995 to 2018 [25,26].

NENs can originate from any organ, but most commonly arise from the gastroenteropancreatic (GEP) and bronchopulmonary tracts [27,28]. These neoplasms secrete peptide hormones and present with a broad spectrum of symptoms based on the hormone secreted [27]. Histologically, NENs can be divided into two major categories: well-differentiated neuroendocrine tumors (NETs) and poorly differentiated neuroendocrine carcinomas (NECs) [20,29]. NETs are further classified into grades G1, G2, and G3 based on their Ki-67 proliferation index and mitotic rate [30]. Despite the availability of highly sensitive diagnostic techniques, a significant portion of histologically confirmed NENs, ranging from 9 to 22%, remains classified as having an unknown primary origin (UPO), due to the absence of a clinically or radiologically identifiable primary site [30]. Owing to their rarity and complex clinical presentation, the study of NENs continues to pose a diagnostic and therapeutic challenge.

**Figure 1:**
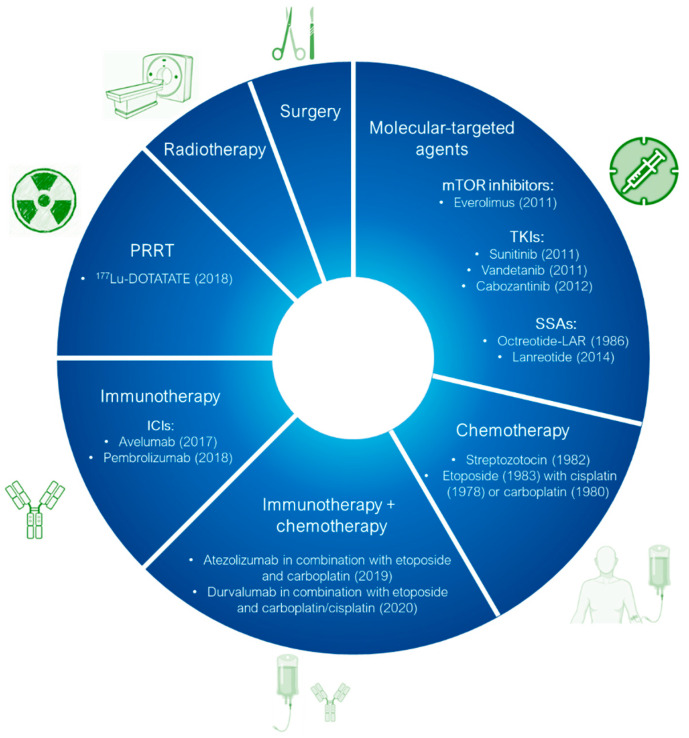
FDA-approved therapies for neuroendocrine neoplasms.

Among the therapeutic options, surgery is the primary choice for resectable NENs. In cases of inoperable, unresectable, or metastatic disease, systemic treatments include radiotherapy, peptide receptor radionuclide therapy (PRRT) (^177^Lu-DOTOTATE), somatostatin analogs (SSAs) (octreotide, lanreotide), mTOR inhibitors (everolimus), tyrosine kinase inhibitors (TKIs) (sunitinib, vandetanib, cabozantinib), immunotherapy (avelumab, pembrolizumab), chemotherapy (streptozotocin, etoposide with cisplatin/carboplatin), and combination therapy (immunotherapy with chemotherapy) (Figure 1). The choice of a specific treatment option depends on the grade and differentiation of the neuroendocrine tumor [20,29,31].

Metallodrugs have had a decisive role in the treatment of NENs, as platinum-based chemotherapy and PRRT are approved for the management of these types of tumors [20]. Over the years, many metallodrugs have also demonstrated promising potential as anticancer drugs [32]. However, the study and development of newer metal-based anticancer agents for NENs treatment remains limited.

## 3. Metal-Based Drugs for NENs Treatment

**Figure 2:**
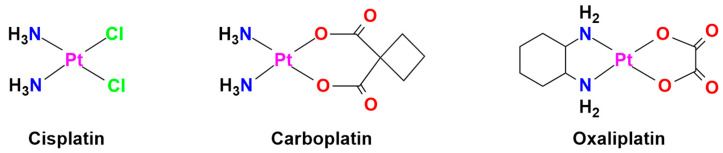
Chemical structures of FDA-approved platinum(II)-based drugs used in cancer therapy.

Metal-based drugs, particularly platinum complexes such as cisplatin, carboplatin, and oxaliplatin, play a pivotal role in cancer treatment protocols (Figure 2). These agents primarily exert their mechanism of action through DNA targeting, inducing cellular damage that ultimately leads to apoptosis [33]. In addition to platinum, ongoing research is investigating the anticancer effects of other metals, including ruthenium, titanium, palladium, and gold, underscoring their potential to broaden the horizon of cancer treatment strategies [34,35]. The standard first-line treatment for advanced neuroendocrine carcinomas (NECs), particularly small cell lung cancer (SCLC)—a poorly differentiated and high-grade NEC [36]—and extrapulmonary NECs (including those in the pancreas, gastrointestinal tract, and others), is a combination of cisplatin or carboplatin with etoposide chemotherapy [31,33,37]. Limitations in the use of platinum-based drugs in NENs include resistance and poor response in well-differentiated neuroendocrine tumors (NETs). Furthermore, current recommendations are derived from small, non-randomized series largely focused on treatment with platinum plus etoposide, with data lacking for other platinum-based regimens [31]. Several studies have demonstrated that oxaliplatin-based regimens (e.g., FOLFOX, CAPOX, GEMOX, XELOX) also have promising antitumor activity in advanced NETs, but the available data are limited [38,39,40,41].

In the design of new metal-based anticancer agents, the gold-based complex, Auranofin, has garnered increasing attention as a potential alternative to traditional cisplatin [42,43]. In a phase I/II study (NCT01737502), Auranofin demonstrated preliminary efficacy in the treatment of neuroendocrine lung cancers when combined with sirolimus, an immunosuppressant. Recently, further in vitro and in vivo studies have shown that Auranofin effectively inhibits thioredoxin reductase (TrxR) in SCLC and sensitizes NETs and SCLC to sorafenib, thus representing an adjuvant therapy with targeted agents that induce disruptions in thiol metabolism [44].

**Figure 3:**
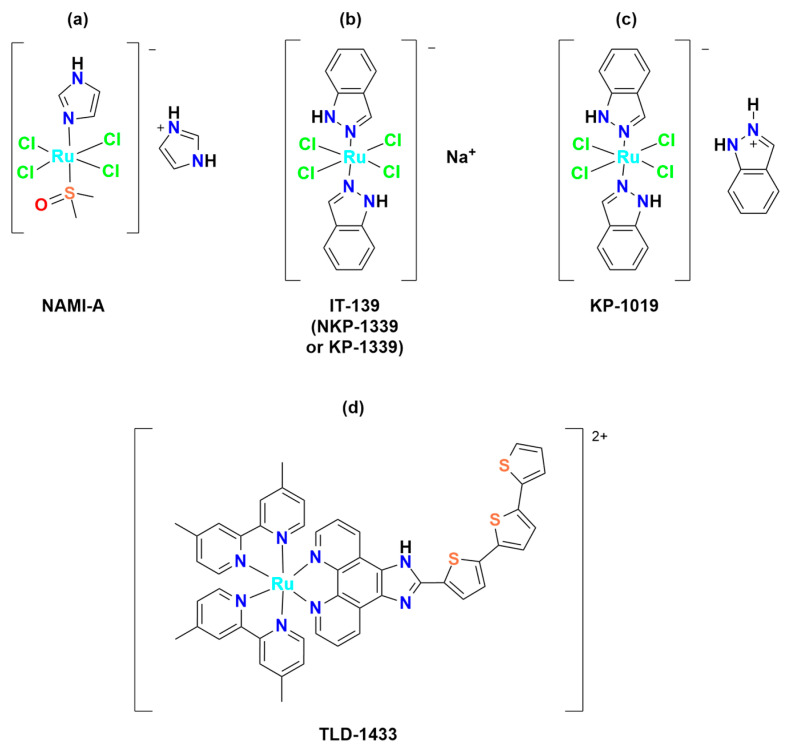
Chemical structures of the ruthenium(III) complexes NAMI-A (**a**), IT-139 (also known as NKP-1339 or KP-1339) (**b**), and the KP-1019 precursor (**c**), as well as the ruthenium(II) complex TLD-1433 (**d**). All of the depicted ruthenium complexes are currently under investigation in clinical trials.

Among the most studied metal-based drugs, ruthenium complexes represent a class of potential chemotherapeutics due to their remarkable antineoplastic and antimetastatic activity [45]. Currently, only three ruthenium(III) complexes, imidazolium *trans*-[tetrachlorido(dimethylsulfoxide)(1H-imidazole)ruthenate(III)] (NAMI-A), [Na]*trans*-[tetrachloro-bis(1H-indazole)ruthenate(III)] (IT-139, NKP-1339 or KP-1339), and indazolium *trans*-[tetrachloridobis(1H-indazole)ruthenate(III)] (KP-1019) (Figure 3a–c) have entered early phase clinical trials for various solid tumors (e.g., colorectal, ovarian, etc.) [46,47,48,49,50,51,52]. A Ru(II) photosensitizer for photodynamic therapy (PDT), TLD-1433 (Figure 3d), has also entered human clinical trials [53,54,55,56]. However, no ruthenium complex is currently in clinical use [54]. Among all these ruthenium complexes, IT-139 (Figure 3b) has attracted particular interest due to its effectiveness against NETs (NCT01415297). BOLD-100 (developed from IT-139) in combination with FOLFOX, showed safety and efficacy results in metastatic colorectal cancer (NCT04421820) [23,57].

In addition to the most extensively studied platinum, ruthenium, and gold complexes, other new metal-based compounds are currently being investigated in vitro and in vivo for their anticancer activity. Pellei and colleagues evaluated the antitumor properties of newly synthesized metal complexes (Mn(II), Fe(II), Co(II), Ni(II), Cu(II), and Zn(II)) against a series of human cancer cell lines derived from various solid tumors, including SCLC [58]. Except for iron derivatives, cellular studies revealed interesting antitumor properties, even against cancer cells with poor sensitivity to traditional cisplatin [58]. Furthermore, three new Ag(I) complexes based on phosphanes and ester derivatives of bis(pyrazol-1-yl)acetate ligands proved to be particularly effective against the highly aggressive and intrinsically resistant human SCLC cells (U1285), thanks to their ability to accumulate in cancer cells and selectively target thioredoxin reductase (TrxR), leading to an imbalance in redox homeostasis and apoptosis [59].

## 4. Ruthenium(II/III) Complexes

Interest in ruthenium(II/III) complexes has grown significantly due to the successes of platinum therapies and the chemical similarities among platinum group metals (Pt, Pd, Rh, Ir, Ru, and Os) [60]. Recently, the chemistry of ruthenium for therapeutic applications has been intensively studied, owing to its advantages over platinum drugs, such as high efficacy also in cisplatin-resistant tumors, low toxicity due to its higher selectivity for cancer cells compared with normal cells, and reduced ability to induce drug resistance operating via alternative mechanisms to platinum drugs [21,22,61,62,63,64]. However, to date, no ruthenium complex is in clinical use [54].

The anticancer activity of ruthenium compounds was first demonstrated in 1976 when *fac*-[Ru(NH_3_)_3_Cl_3_] exhibited similar cytotoxic effects to cisplatin in a few selected cell lines [65,66,67]. Since then, several ruthenium-based complexes have been synthesized and reported for their anticancer properties [68]. They have also been identified as potential antimicrobials, antimalarials, immunosuppressants, nitric oxide scavengers, and antivirals [69,70,71,72,73].

Ruthenium, with its variable oxidation state ranging from +2 to +8, serves as a unique catalyst in oxidative reactions. Ruthenium compounds primarily exist in three oxidation states. The high oxidation state of Ru(IV) compounds is unstable, limiting their further development [22,74]. Instead, Ru(II) and Ru(III) coordination compounds exhibit excellent activity against cancer. Due to their higher effective nuclear charge, Ru(III) complexes are more inert than Ru(II), being able to act as prodrugs [75,76]. Tumor cells have a more chemically reducing environment than normal healthy cells (hypoxia, acidic pH, and high glutathione levels). Thus, Ru(III) can be taken up in the relatively inert oxidation state, causing minimal damage to healthy cells, and reduced to its corresponding (active) Ru(II) counterpart in vivo [75,76,77,78,79]. Based on their scaffolds, the Ru complexes studied for anticancer activity can be categorized into two main groups: mononuclear complexes (e.g., arene, half-sandwiched or piano-stool, and cyclometalated complexes) and multinuclear complexes (homonuclear and heteronuclear) [68,80]. The characteristics of these compounds allow Ru-based drugs to target multiple sites and employ various mechanisms of action to induce cancer cell death [22].

Among the Ru(III)-based drugs that have progressed to clinical studies, NAMI-A (Figure 3a) showed the highest antimetastatic effects. However, phase II clinical studies demonstrated that it caused severe side effects, thus further investigations were halted. Also, the further tested KP-1019 (Figure 3c) demonstrated unsatisfactory efficacy for clinical study due to severe side effects and poor water solubility. To overcome these problems, the more soluble sodium salt IT-139 (Figure 3b) was designed and is currently being used in clinical studies.

**Figure 4:**
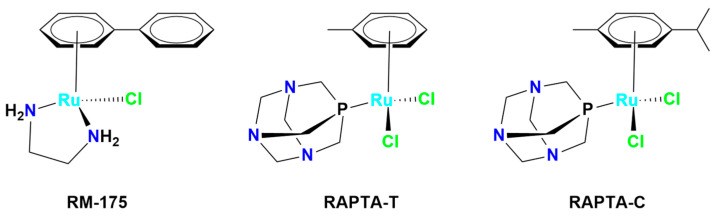
Chemical structures of ruthenium(II)-based complexes in pre-clinical trials.

In addition to Ru(III), several Ru(II)-arene complexes, such as RM-175 and 1,3,5-triaza-7-phosphaadamantane (PTA)-containing complexes (RAPTA-C and RAPTA-T) (Figure 4), were submitted to advanced pre-clinical studies [34,81,82,83,84,85,86,87,88]. Interest in Ru(II) coordination complexes also arose due to their photophysical properties, which include increased excited state lifetime, efficient ^1^O_2_ production, visible light absorption, high cellular uptake, and two-photon excitation [89]. Many Ru(II) compounds showed better antitumor activities than their corresponding Ru(III) counterparts in vivo [22].

Presently, the progression of ruthenium antitumor agents is transitioning from singular treatment modalities to combination therapies [22,54,90,91]. The integration of diverse approaches (including chemotherapy, radiotherapy, photodynamic therapy, photothermal therapy, immunotherapy, and gene therapy) results in a synergistic effect that significantly enhances therapeutic efficacy. Furthermore, this comprehensive strategy addresses prevalent challenges associated with monotherapy, such as drug resistance, tumor recurrence, and systemic toxicity [54].

### 4.1. Mechanisms of Action

Ruthenium complexes are administered through intravenous injections. In the blood, these drugs, like other metallotherapeutics, can be transported by albumin and transferrin [54,92]. Based on current knowledge, the uptake of ruthenium complexes could depend on more than one mechanism. It has been demonstrated that they can enter cells by simple or facilitated diffusion (e.g., KP-1019) and possibly by active transport mechanisms, though these are not yet fully elucidated [54]. Furthermore, endocytosis plays an important role in Ru complex uptake, especially receptor-mediated endocytosis, which also increases tumor targeting [54,93,94]. Transferrin/transferrin receptor (TfR)-mediated endocytosis (mediated by clathrin) is one of the modes of endocytosis for Ru compounds [54,95,96]. The transferrin binding and cellular ruthenium uptake may depend on the nature of the Ru complex, the availability of Fe(III) binding sites of transferrin, and the presence of competing proteins which bind metal, such as serum albumin [94]. The iron transport protein transferrin is of significant interest due to the increased demand for iron in tumors, resulting in the overexpression of transferrin receptor 1 (TfR1) [52,97,98]. Some studies have demonstrated that TfR also serves as a marker of the malignant phenotype in neuroendocrine carcinomas (NEC) [99,100,101]. As a result, NE tumor cells could be specifically targeted by Ru-based drugs that bind to transferrin [52,97,98,102].

**Figure 5:**
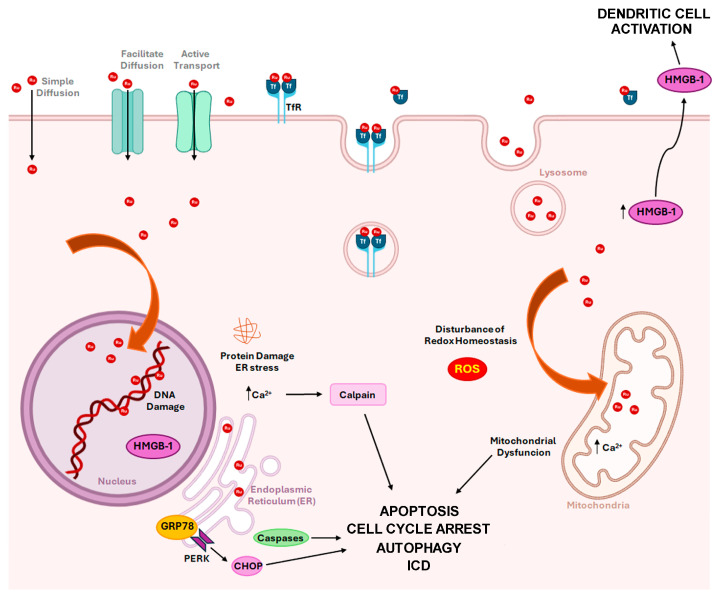
Proposed mechanism of action of Ru(III) complex IT-139 (NKP-1339 or KP-1339). Ru complexes can enter cells by simple diffusion, facilitated diffusion, active transport, or transferrin (Tf)-mediated uptake. Their antitumoral action appears to be related not only to DNA damage and ROS induction but primarily to the disruption of endoplasmic reticulum (ER) homeostasis by depletion of key cellular chaperons, including GRP78, in combination with enhanced IT-139-mediated protein damage. Induction of cell death could occur through the PERK/eIF2α/ATF4/CHOP signaling pathway, calpain, and caspase activation. Moreover, IT-139 treatment could lead to upregulation of the mitogen-activated protein kinase (MAPK) stress response pathway and accumulation of HMGB-1 in the cytoplasm and extracellular space. All these effects ultimately lead to apoptosis, cell cycle arrest in the G2/M phase, autophagy activation, and ICD.

Generally, during endocytosis, external molecules are internalized by the cellular membrane to form endosomes, which can reach lysosomes [54]. From lysosomes, Ru complexes can then translocate to other compartments, such as the nucleus, mitochondria, and endoplasmic reticulum [54,102] (Figure 5). Therefore, they can interact with a variety of cellular targets involving numerous chemical strategies rather than only DNA interactions [60]. Some studies have revealed that mitochondria are a key target of Ru complexes [103,104,105]. Mitochondria of cancer cells differ structurally and functionally from their normal counterparts in several properties, such as their higher negative mitochondrial membrane potential and metabolic reprogramming [106,107]. Positively charged Ru complexes may be localized in mitochondria due to the negative potential of the mitochondrial inner membrane attracting lipophilic cations, including charged metal compounds (e.g., polypyridyl Ru complexes) [108,109,110]. Mitochondria-accumulating Ru(II) complexes can induce cancer cell death by depleting energy generation and inducing oxidative stress via disturbing intracellular iron content and glutathione (GSH) levels [107]. Moreover, they can activate mitochondrial dysfunction by regulating Bcl-2 family proteins, leading to intracellular ROS (reactive oxygen species) overproduction [105,111,112]. At the same time, nuclear permeable Ru compounds can induce genomic DNA damage, ultimately activating apoptosis, necrosis, autophagy-dependent cell death, and cell cycle arrest, and inhibit angiogenesis, thus leading to the blockage of tumor growth and metastasis [54,112,113,114] (Figure 5).

### 4.2. The Case of IT-139

The Ru(III) complex IT-139 (KP-1339 or NKP-1339) (Figure 3b) completed phase I trials for solid tumors treatment (NCT01415297), showing good antitumor activity in patients with carcinoid NETs, including those resistant to different classes of anticancer agents [23,57]. The IT-139 complex has some structural similarity to NAMI-A due to its octahedral geometry and chloride ligands, but it is a pro-drug that is relatively inert compared to NAMI-A, as ligand loss or exchange does not occur as readily [60]. In terms of both antitumor activity and side effects, IT-139 exhibits a distinct pre-clinical and clinical profile compared to NAMI-A, which likely arises from its novel mechanism of action [23] (Figure 5). The formation of a metal–blood protein adduct accounts for the low side effect profile of IT-139 (about 625 mg/m^2^ was shown to be the highest dose that could be tolerated), as this Ru(III) complex remains in its pro-drug form until it undergoes “activation by reduction” [49,51,52,60,115] to form more active Ru(II) species [116]. The slow ligand exchange rate of IT-139 and the lack of correlation between its efficacy and p53 and KRAS mutations (in contrast with cisplatin) imply that DNA binding may not be the primary mechanism of action of this Ru(III) complex [117]. This may explain why IT-139. which shows activity against platinum-resistant cell lines in pre-clinical studies [60,117,118]. The promising antitumor activity of IT-139 is also associated with its immunogenic effects and its ability to alter glycolysis in cancer cells [94]. The whole genome expression arrays demonstrated that responsiveness to IT-139 may be related to specific gene expression alterations and to protein damage. IT-139-sensitive cell lines upregulated genes that are involved in the response to chemical stimuli, regulation of cell death, and chromatin organization, while low-responsive cells showed deregulation of genes involved in cell cycle and metabolism [117].

The primary antitumor effects of IT-139 are attributed to suppression of the 78-kDa glucose-regulated protein (GRP78), also known as the immunoglobulin heavy chain-binding protein (BiP). GRP78 is a key regulator of the unfolded protein response (UPR), promoting protein folding and assembly, controlling protein quality, and regulating endoplasmic reticulum (ER) stress-signaling pathways that lead to UPR survival and apoptosis responses. Upon ER stress, when misfolded proteins accumulate in the ER, GRP78 binds to these proteins, releasing and activating three ER transmembrane signal transducers: protein kinase RNA-like endoplasmic reticulum kinase (PERK), inositol-requiring enzyme 1 (IRE1), and activating transcription factor 6 (ATF6). This leads to the induction of UPR [119,120,121]. Additionally, activated PERK phosphorylates NRF2, generating an antioxidant response that inhibits the accumulation of reactive oxygen species (ROS) [121].

GRP78 can be overexpressed in cancer, functioning as a central stress sensor that detects and adapts to changes in the tumor microenvironment. Stress conditions such as hypoxia, glucose deficiency, and lactic acidosis can induce cell surface GRP78 expression in cancer cells. This activates the unfolded protein response (UPR) signal, enabling tumor cells to adapt to the microenvironment of the metastatic site and acquire the ability to survive and establish at the metastatic site [121,122,123,124]. Chemotherapy drugs can also induce cell surface GRP78 expression, leading to chemoresistance [125]. It has been observed that upregulation of GRP78 confers higher resistance to cisplatin. Conversely, reducing GRP78 levels could enhance cell death and re-sensitize cisplatin-resistant cells in vitro [126].

IT-139 is able to suppress GRP78 expression in a wide range of cancer cells via multiple mechanisms, with minimal effect on GRP78 stress induction in normal human cell lines and primary cells [23,118,123].

Moreover, IT-139 alters intracellular calcium ion (Ca^2+^) homeostasis leading to the activation of calpain (a non-lysosomal cysteine protease) and to upregulation of the p38 mitogen-activated protein kinase (MAPK) stress response pathway [127,128,129].

Interestingly, IT-139 represents the first recognized ruthenium-based complex that is likely able to trigger immunogenic cancer cell death (ICD) in vitro (Figure 5). ICD induction is related to the PERK/eIF2a/ATF4/CHOP signaling pathway and to the activation of caspases, such as caspase-3, 7 and 8 [124,129,130,131,132]. Chemotherapy-induced ICD by IT-139 was demonstrated by the emission of key immunomodulatory damage-associated molecular patterns (DAMPs), such as pre-apoptotic calreticulin (CRT) surface exposure, extracellular ATP (adenosine triphosphate), and high mobility group box 1 (HMGB-1) [124]. HMGB-1 is normally localized in the nucleus and under homeostatic conditions can shuttle between the nucleus and the cytoplasm. Its secretion or passive release through permeabilized plasma membrane constitutes a major cellular danger signal. In the extracellular space, it binds to toll-like receptor 4 (TLR-4) on the surface of dendritic cells to optimize the presentation of antigens from dying tumor cells [124,133]. Finally, the ATP release during IT-139-induced ICD leads to the activation of the autophagic process (upregulation of Beclin-1 and LC3A/B-II) [117,124,129,134,135] (Figure 5).

### 4.3. New Ruthenium-Based Drug in Pre-Clinical Studies

The field of Ru-based agents has yielded some very promising anticancer agents with exceptional in vivo and in vitro activity [114,136]. The intense investigations on ruthenium complexes over the last thirty years have drawn a lot of attention to these compounds due to their potential as selective antimetastatic drugs with low systemic toxicity [49,137]. Despite the interesting discovery of IT-139 efficacy against neuroendocrine neoplasms [23], few studies have investigated Ru-based complexes in these tumors. Some pre-clinical works have highlighted interesting results in terms of antitumor capabilities of Ru(II) [46,138,139,140] and Ru(III) [141] complexes against neuroendocrine (NE) in vitro models (Table 1).

**Figure 6:**
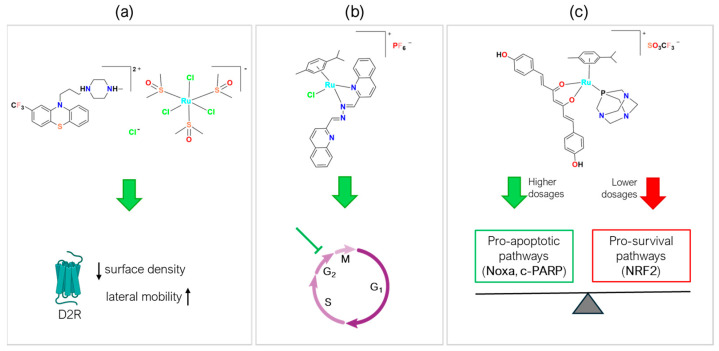
Structures of novel experimental Ru compounds. The chemical structure of new Ru-based complexes (**a**) (TF·H_2_)[RuCl_3_(DMSO)_3_]Cl·0.5C_2_H_5_OH [139]; (**b**) [(η^6^-p-cym)(2-bis(quinolin-2-ylmethylene)hydrazine)RuCl]PF_6_ [46], and (**c**) [(cym)Ru(bdcurc)(PTA)]SO_3_CF_3_ [138]) and their effects on NE cancer cell lines are reported. (**a**) (TF·H2)[RuCl_3_(DMSO)_3_]Cl reduces dopamine D2 receptors’ surface density and increases their lateral mobility; (**b**) pCYRuL demonstrates high selectivity (submicromolar activity) against cancer cells and induces cell cycle arrest, primarily in the G2/M phase; (**c**) Ru-bdcurc treatment induces pro-apoptotic (PARP cleavage, Noxa expression) pathways at higher doses or cell-death-resistant pathways (NRF2-induced targets, 4E-BP1, Bcl-2 and p53) at lower doses.

Leskovac et al. (2018) investigated the anticancer properties of three Ru(II)-N-alkyl complexes, each with a different phenothiazine (chlorpromazine hydrochloride, thioridazine hydrochloride, or trifluoperazine dihydrochloride) as counterion [139]. Phenothiazines, which modulate a variety of neurotransmitter activities, including dopaminergic and cholinergic signaling, have been identified as potential in vitro anticancer agents [143,144]. They can induce oxidative stress and mitochondrial dysfunction, leading to cell death [139,145]. In addition, the high hydrophobicity of phenothiazines favors interaction with cellular membranes and may contribute to the cytotoxicity of the complex [139,145]. In vitro kinetic investigations showed the capability of this type of Ru compound to inhibit acetylcholinesterase (AChE), although to a lesser extent compared to corresponding counterions [139]. Conversely, the cytotoxicity of these Ru complexes seemed higher than that of their corresponding counterions [146]. The antiproliferative effects of these compounds were evaluated in several cell lines [146], including neuroendocrine PC-12 cells (Table 1; Figure 6a). Complexes with chlorpromazine and thioridazine demonstrated potent cytotoxic and pro-oxidant actions compared to the complex with trifluoperazine as a counterion. However, no data regarding this has been reported for the neuroendocrine PC-12 cell line [139].

To the best of our knowledge, the study by Leskovac et al. is the only one that has considered the in vitro role of dopamine D2 receptors (D2R) in NE cancer cells following treatment with a Ru compound. Given that phenothiazines can affect D2R, Leskovac and colleagues evaluated the influence of the Ru complexes on D2R distribution in the plasma membrane of rat neuroendocrine PC-12 cells (Table 1). Although these Ru(II)-N-alkyl phenothiazine complexes have been bound to D2R, only the complex containing trifluoperazine dihydrochloride, (TF·H_2_)[RuCl_3_(DMSO)_3_]Cl·0.5C_2_H_5_OH, reduced its surface density and increased its lateral mobility. This could be due to direct action on D2R or indirectly through alternative targets, making it a promising candidate drug for the treatment of cancers that overexpress D2R [139] (Table 1; Figure 6a).

Recently, Khan and colleagues investigated the antiproliferative effect of a family of Ru(II) complexes with a half-sandwich structure and a new bis-quinoline-based ligand. These complexes were highly active against PC-3 [46,142], a castration-resistant prostatic adenocarcinoma cell line that is positive for neuroendocrine markers [147]. All Ru complexes were found to be more cytotoxic than cisplatin, indicating some selectivity likely due to the nature of PC-3 cells. These complexes demonstrated a high affinity for double-stranded DNA, which could potentially explain the observed anticancer activities [142]. A structure–activity comparison revealed that the planarity of the molecules could facilitate intercalation within DNA base pairs. However, substitution with a bulky alkyl group might interfere with intercalation but could contribute to other type of interactions (Table 1). Among the tested complexes, the [(η^6^-*p*-cymene)(2-bis(quinolin-2-ylmethylene)hydrazine)RuCl]PF_6_ complex (pCYRuL) was demonstrated to be more effective (submicromolar activity) against PC-3, probably due to the different permeability of these cells. Furthermore, pCYRuL did not demonstrate significant toxicity in normal human cells. In the neuroendocrine prostatic cancer cells, this compound induced cell cycle arrest, mainly in the G2/M phase, indicating that pCYRuL interacts with DNA and causes DNA damage [46] (Table 1; Figure 6b).

Differently from Leskovac [139] and Khan [46,142], the anticancer effects of Ru(II) complex [(cymene)Ru(bdcurc)(PTA)]SO_3_CF_3_ (PTA = 1,3,5-triaza-7-phosphaadamantane; bdcurc = bisdemethoxycurcumin) [148] were more deeply examined by Garufi et al. using the BON-1 cell line—one of the few cell lines derived from GEP-NEN (Table 1; Figure 6c). In vitro, the compounds demonstrated a dose-dependent induction of cell death. Higher Ru-compound doses induced pro-apoptotic pathways (e.g., PARP cleavage, Noxa expression). On the other hand, lower treatment dosages caused activation of cell-death-resistant pathways, such as erythroid 2-related factor 2 (NRF2)-induced targets (catalase and p62), Bcl-2, and p53 (which is dysfunctional in BON-1 cells) (Figure 6c). The genetic or pharmacological block of the p53/NRF2 pathway enhanced cytotoxic effects in response to lower dosages of Ru-bdcurc. Furthermore, lower Ru-complex dosage caused the phosphorylation of 4E-BP1, an mTOR target whose activation can sustain cancer cell survival and predict poor prognosis. The hyperphosphorylated 4E-BP1 was correlated to a poor prognosis in patients with different types of tumors [149,150,151,152,153].

Finally, D’Amora (2019) studied the chemical and biological behavior of two NAMI-A-class sugar-incorporated Ru(III) complexes, (Na[*trans*-RuCl_4_(pyridine)(DMSO)] (RuPy) and Na[*trans*-RuCl_4_(PyTry)(DMSO)] (RuPyTry) (PyTry = 1,4-disubstituted-1,2,3-triazole)) in PC-3 cells. The presence of the fully sugar-protecting group in the second compound did not enhance the cytotoxic activity when compared to RuPy, and both complexes demonstrated very low cytotoxicity. However, their encapsulation in liposomes resulted in comparable or higher cytotoxic effects compared to cisplatin and a higher selectivity toward the malignant cells in the case of lipo-RuPyTry preparation (Table 1). This may be due to the overcoming of membrane-crossing problems and the relative amount of water in the inner liposome compartment, which made the ligand kinetic exchange of chlorido ligands slower [141].

## 5. Ru Complexes Targets and NENs: New Possible Therapeutic Strategies

Over the past few decades, the role of specific biomarkers that can predict the response of neuroendocrine neoplasms (NENs) to platinum-based drugs has been extensively studied [20]. However, due to the heterogeneity of these neoplasms, a better understanding of the mechanisms underlying different treatment outcomes, which are often unfavorable, is required. Like platinum drugs, ruthenium-based agents can interact with a variety of cellular targets using different chemical strategies not limited to DNA interactions, as summarized in Table 2 [60,154]. However, their mechanism of action is even less understood than that of traditional platinum compounds.

IT-139, as well as other Ru complexes, act by inhibiting GRP78 [117,148] and overcoming GRP78-mediated chemoresistance [123]. Consistently, key players in endoplasmic reticulum (ER) stress often appear to significantly contribute to the progression of neuroendocrine neoplasms (NENs). The upregulation of GRP78, along with ATF4 and CHOP, has been observed in pancreatic NET patients compared to controls [121,125,155,156,157] (Figure 5). GRP78 is widely involved in the induction of tumor proliferation, metastasis, drug resistance, and apoptosis, representing an adaptive response that allows cells to overcome proteotoxic stress. Since GRP78 upregulation in tumor cells is associated with intrinsic and drug-induced resistance, it is considered a good marker to predict the response to therapy. Its inhibition increases the sensitivity of cancer cells to chemotherapy-induced apoptosis [121,125,155,156,157].

**Table 2:** Molecular and structural formulas of the mentioned ruthenium(II) and ruthenium(III) complexes in the clinical and pre-clinical phase and their general mechanism of action.

Drug and Molecular Formula	Chemical Structure	Mechanism of Action	Study Phase
**Ru(II) Compounds**			
TLD-1433 [Ru(4,4′-dimethyl-2,2′-bipyridine)_2_(2-(2′,2″:5″,2′′′-terthiophene)-imidazo [4,5-f][1,10]phenanthroline)]^2+^	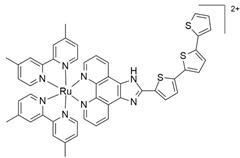	ROS generation by photodynamic therapy (PDT) and photochemotherapy (PCT) [53,55,56]	Phase II
RM-175 [Ru(η^6^-biphenyl)Cl(ethylenediamine)]PF_6_	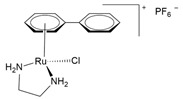	DNA covalent interaction or non-covalent interaction (intercalation) [86]	Pre-clinical study
RAPTA-C [Ru(η^6^-p-cymene)Cl_2_(1,3,5-triaza-7-phosphaadamantane)]	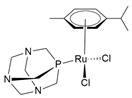	Protein alteration; adducts at specific histone sites or the DNA components [158]	In vitro and in vivo pre-clinical study
RAPTA-T Ru(η^6^-toluene)Cl_2_(1,3,5-triaza-7-phosphaadamantane)	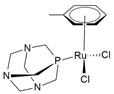	Cytoskeleton alteration; histone modification [87,88]	In vitro and in vivo pre-clinical study
(chlorpromazine) [RuCl_3_(DMSO)_3_]·C_2_H_5_OH (thioridazine) [RuCl_3_(DMSO)_3_]·0.5C_2_H_5_OH (trifluoperazine) [RuCl_3_(DMSO)_3_]Cl· 0.5C_2_H_5_OH	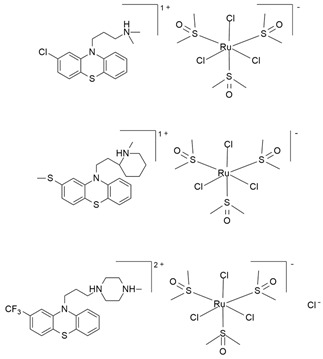	Not identified [139,146]	In vivo and in vitro pre-clinical study
Ru-bdcurc [(cymene)Ru(bisdemethoxycurcumin)(1,3,5-triaza-7-phosphaadamantane)]SO_3_CF_3_	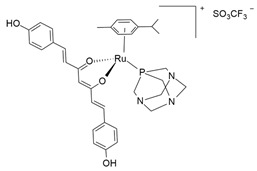	DNA damage (unclear) [138,148]	In vitro pre-clinical study
pCYRuL [(η^6^-*p*-cymene)(1,2-*bis*(quinolin-2-ylmethylene)hydrazine)RuCl]PF_6_ BzRuL [(η^6^-benzene)(1,2-*bis*(quinolin-2-ylmethylene)hydrazine)RuCl]PF_6_ HmbRuL [(η^6^-hexamethylbenzene)(1,2-bis(quinolin-2-ylmethylene)hydrazine)RuCl]PF_6_	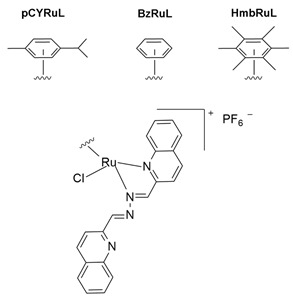	DNA damage [46,142]	In vitro pre-clinical study
**Ru(III) Compounds**			
NAMI-A *trans*-[tetrachlorido(dimethylsulfoxide)(1H-imidazole)ruthenate(III)]	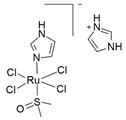	DNA and RNA damage [48,49,50]	Phase II
IT-139 or NKP-1339 or KP-1339 [Na]*trans*-[tetrachloro-bis(1H-indazole)ruthenate(III)]	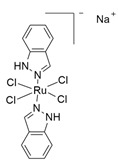	DNA damage; ROS generation; ER stress (GRP78) [51,52]	Phase I
KP-1019 indazolium *trans*-[tetrachloridobis(1H-indazole)ruthenate(III)]	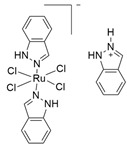	DNA damage; ROS generation; osmotic stress [49]	Phase I
*fac*-[Ru(NH_3_)_3_Cl_3_]	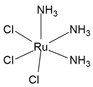	DNA damage [65]	In vitro pre-clinical study
RuPy Na[*trans*-RuCl_4_(pyridine)(DMSO) RuPyTry Na[*trans*-RuCl_4_(1,4-disubstituted-1,2,3-triazole)(DMSO)]	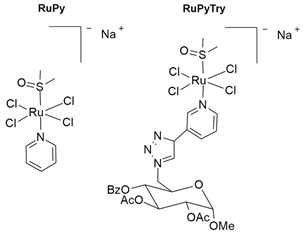	Not identified [141]	In vitro pre-clinical study

Among the ruthenium-based complexes considered in this review for their application in NEN models in vitro, the [(cymene)Ru(bdcurc)(PTA)]SO_3_CF_3_ (Ru-bdcurc) complex is the one for which the mechanism of action has been most extensively studied, although its mechanism of action still needs to be clarified [138,148] (Table 1; Figure 6c). Garufi et al. (2023) highlighted the negative effect of GRP78 and NRF2 on Ru-bdcurc cytotoxicity in colon cancer cells, as its use led to an increase in the levels of these two molecules [148]. Unfortunately, the role of GRP78 in this Ru complex’s mechanism of action was not evaluated in pancreatic neuroendocrine BON-1 cells [138]. However, NRF2 appeared to be implicated in both tumor cell types, with reduced cytotoxicity [138,148]. NRF2 is a transcription factor that regulates cellular antioxidant and stress responses, driving cancer progression, metastasis, and resistance to therapy [159]. Increased NRF2 levels were found in colon cancer cells after treatment with both lower and higher dosages of Ru-bdcurc, along with a consistent increase in GRP78 levels [148]. Increased NRF2 levels were found in BON-1 cells only after treatment with a lower dosage of the Ru compound. Thus, the NRF2 resistance mechanism to the Ru-bdcurc complex appeared less pronounced in cancer cells of neuroendocrine origin [138]. Gurufi et al. suggest that the treatment outcome is achieved not only through the antioxidant effect of NRF2, but also because NRF2 collaborates with several different oncogenic pathways. In fact, pathways that are interconnected and/or act in a feedback loop with NRF2 can lead cancer cells to adapt to stresses induced by anticancer therapies [138,148,160].

Recently, the role of oxidative stress has been studied in pituitary NET, in which NRF2 is highly expressed [160,161,162,163,164]. Some studies have revealed that the NRF2 pathway is activated in pituitary tumors and is associated with mitochondrial dysfunction and the modulation of oxidative stress-regulated immune cells in the tumor microenvironment (TME) [160,164]. Therefore, potential drugs for the treatment of these types of NETs should preferably act via NRF2-mediated redox modulation. Relevant to gastroenteropancreatic neuroendocrine neoplasm (GEP-NEN) research, Garufi et al. identified, for the first time, a possible dysfunction of the NRF2 pathway in BON-1 cells. This reveals an important factor in cell resistance to cytotoxic drugs, including neuroendocrine cancer cells [138] (Table 1; Figure 6c).

In addition to GRP78 and NRF2, phenothiazines have also been associated with oxidative stress and mitochondrial dysfunction (Figure 6a). Recently, mitochondria-targeted photodynamic therapy based on phenothiazine demonstrated enhanced immunogenic cancer cell death (ICD) via mitochondrial oxidative stress in vivo [165]. Interestingly, the GRP78/PERK/eIF2α signaling pathway of the ER induced by IT-139 plays an essential role in the cascade of events triggering ICD [124] (Figure 5). Recently, the induction of ICD was demonstrated for several Ru complexes in pre-clinical studies [166,167,168,169,170].

Derivatives of phenothiazine could act on cells through the antagonism of dopamine D2 receptors (D2R). Recent data have demonstrated that D2R are expressed in NETs, and successful trials of the NET treatment with dopamine agonists have been reported [171,172,173]. Recently, Kayum Alam and colleagues demonstrated that D2R agonists sensitize chemotherapy-resistant neuroendocrine tumors to cisplatin and etoposide, reducing tumor angiogenesis in multiple in vivo xenograft models of human small cell lung cancer (SCLC) [174]. These results have clinical relevance considering the potential role of dopaminergic drugs in inhibiting secretion and/or cell proliferation in NETs [172,174,175,176]. Furthermore, the antiangiogenic properties of anticancer agents, including Ru compounds, may be of significant interest in NETs, as they are typically highly vascularized tumors [177].

## 6. Conclusions and Perspectives

Over the past few decades, various anticancer metallodrugs have emerged as agents for controlling compound reactivity and stability in physiological environments. Among the most studied metal-based complexes, ruthenium complexes represent the most promising alternatives to platinum-based anticancer agents due to their multifunctional biochemical properties.

Neuroendocrine neoplasms (NENs) represent a small group of malignancies with diverse prognosis, biology, and behavior. Probably, the relative rarity of this type of malignancies results in a lack of studies in the literature aimed at studying metallodrugs and, in general, chemotherapy agents in NENs. Currently, some ruthenium complexes are undergoing clinical trials for the treatment of solid tumors, including neuroendocrine neoplasms (Table 2). The Ru(III) complex IT-139 is particularly interesting, since in clinical trials it demonstrated higher antitumor activity in patients with carcinoid NETs, including those resistant to different classes of anticancer agents. While it would be interesting to evaluate its selectivity in certain types of tumors, despite numerous investigations, the mechanisms of action of IT-139 remain largely unclear. In addition, the identification of specific biomarkers would be desirable in order to predict therapeutic outcomes in patients with NENs after treatment with IT-139. Some pre-clinical studies have highlighted interesting results in terms of the antitumor capabilities of Ru(II) and Ru(III) complexes against NENs, confirming or identifying new potential therapeutic targets and pathways involved in Ru-compound treatment resistance (Table 2). The high cytotoxicity and selectivity against NE cells demonstrated by some of these new Ru compounds should encourage further studies to identify new complexes that can improve specificity and reduce side effects. The study of the mechanisms of action of ruthenium complexes often reveals the involvement of specific pathways, such as GRP78/NRF2, which can either lead to cell death or drug resistance, depending on the features of the tumor cell and the characteristics of the metallodrug. Given the importance of these pathways in NENs, further studies would be useful for the scientific community to identify the advantages of using a specific class of antitumor agents. Furthermore, the emergent role of Ru compounds in immunogenic cancer cell death (ICD) induction should lead to deeper study of this regulated form of cell death in Ru-based-drug-sensitive neoplasms. This also concerns NENs, on which no work on the role of ICD is currently present in the literature.

## Figures and Tables

**Table 1:** New Ru(II)- and (III)-based anticancer complexes in neuroendocrine (NE) cancer models (PC-12, rat pheochromocytoma; PC-3, human prostate adenocarcinoma; BON-1, human pancreatic tumor).

Ru Complexes	NE Cancer Cell Lines	Biological Effects	References
(CP·H)[RuCl_3_(DMSO)_3_]·C_2_H_5_OH (CP·H = chlorpromazine); (TR·H)[RuCl_3_(DMSO)_3_]·0.5C_2_H_5_OH (TR·H = thioridazine); (TF·H_2_)[RuCl_3_(DMSO)_3_]Cl·0.5C_2_H_5_OH (TF·H_2_ = trifluoperazine).	PC-12	Ru(II) compounds can reduce cell viability in NE cancer cells transfected to express dopamine D2 receptor.	[139]
Na[*trans*-RuCl_4_(pyridine)(DMSO)] (RuPy) encapsulated or not in liposome; Na[*trans*-RuCl_4_(PyTry)(DMSO)] (RuPyTry) (PyTry = 1,4-disubstituted-1,2,3-triazole) encapsulated or not in liposome.	PC-3	The pyridine Ru(III) complexes as free drugs do not show significant cytotoxic effects. Instead, RuPyTry and RuPy incorporated in liposomes are equally or more cytotoxic than cisplatin (IC_50_ = 8.0 μM), respectively. The lipo-RuPyTry preparation results are more selective against cancer cells.	[141]
[(cymene)Ru(bdcurc)(PTA)]SO_3_CF_3_ (bdcurc = bisdemethoxycurcumin; PTA = 1,3,5-triaza-7-phosphaadamantane) (Ru-bdcurc).	BON-1	Ru-bdcurc compound induces cell death in a dose-dependent manner, in vitro (EC_50_ = 100 μM). NRF2 activation reduces the cytotoxic effects of the compound.	[138]
[(η^6^-*p*-cymene)(1,2-*bis*(quinolin-2-ylmethylene)hydrazine)RuCl]PF_6_ (pCYRuL); [(η^6^-benzene)(1,2-*bis*(quinolin-2-ylmethylene)hydrazine)RuCl]PF6 (BzRuL); [(η^6^-hexamethylbenzene)(1,2-bis(quinolin-2-ylmethylene)hydrazine)RuCl]PF6 (HmbRuL).	PC-3	The Ru complexes exhibit higher cytotoxicity compared to cisplatin, especially *p*-cymene-containing compounds. Among the tested cell lines (PC-3, A0287, MCF-7, PANC-1), pCYRuL complex has higher antitumor activity against PC-3 (IC_50_ = 0.71 μM), compared to cisplatin (IC_50_ = 31.3 μM), and it is non-toxic in normal human cell lines (HK2 and MCF10A). In PC-3, pCYRuL arrests the cell cycle in the G2/M phase.	[46,142]

## Data Availability

No new data were created or analyzed in this study. Data sharing is not applicable to this article.

## Abbreviations

AChE	Acetylcholinesterase
ATF	Activating Transcription Factor
BiP	Immunoglobulin Heavy Chain-Binding Protein
CHOP	C/EBP Homologous Protein
D2R	Dopamine Receptor 2
GEP	Gastroenteropancreatic
GRP78	Glucose-Regulated Protein
ICD	Immunogenic Cell Death
IRE1	Inositol-Requiring Enzyme 1
MAPK	Mitogen-Activated Protein Kinase
mTOR	Mammalian Target Of Rapamycin
NEC	Neuroendocrine Carcinoma
NEN	Neuroendocrine Neoplasm
NET	Neuroendocrine Tumor
PDT	Photodynamic Therapy
PERK	Double-Stranded RNA-Activated Protein Kinase (PKR)-Like ER Kinase
PRRT	Peptide Receptor Radionuclide Therapy
SCLC	Small Cell Lung Cancer
SSA	Somatostatin Analog
TfR	Transferrin Receptor
TKI	Tyrosine Kinase Inhibitors
TME	Tumor Micro-Environment
TrxR	Thioredoxin Reductases
UPR	Unfolded Protein Response
